# A genetic survival network for glioblastoma multiforme

**DOI:** 10.1186/gb-2011-12-s1-p21

**Published:** 2011-09-19

**Authors:** Andy Lin, Desmond J Smith

**Affiliations:** 1 Department of Molecular and Medical Pharmacology, David Geffen School of Medicine, University of California, Los Angeles, CA 90095, USA

## Background

Most studies exploring cancer progression have focused on the influence of individual genes, and few efforts have investigated the effects of interactions between genes within the genome. Our hypothesis is that cancer cells thrive by exploiting combinations of genes, in fact by exploiting networks of genes that both protect the cell against destruction and enhance its survival. We believe that these networks involve genes that tend to be coordinated in their copy number alterations, even when they are located at a distance in the genome. Radiation hybrid (RH) cells have a random assortment of genes as triploid rather than diploid. Our recent work studying genetic networks in libraries of RH cells has elucidated key survival-enhancing interactions with high specificity [1]. Because of the hardiness of the RH clones, statistically significant patterns of co-inherited, unlinked triploid gene pairs pointed to the cell survival mechanism. We identified more than 7.2 million significant interactions at single-gene resolution using the RH data.

## Methods

Our work with the RH data provided the rationale for an investigation of cancer survival networks, in particular for glioblastoma multiforme, a formidable brain cancer for which extensive datasets are available but few treatment options. We investigated correlated patterns of copy number alterations for distant genes in glioblastoma multiforme tumors using the same method we employed to construct the RH survival network. Public data were analyzed from 301 glioblastomas that had been assessed for copy number alterations using array comparative genomic hybridization [2].

## Results

The glioblastoma and RH survival networks overlapped significantly (*P* = 3.7 × 10^–31^). We therefore exploited the high-resolution mapping of the RH data to obtain single-gene specificity in the glioblastoma network. The combined network features 5,439 genes and 13,846 interactions (false discovery rate (FDR) <5%) and suggests novel approaches to therapy for glioblastoma. For example, although the epidermal growth-factor receptor (*EGFR*) oncogene is frequently activated in glioblastoma, EGFR inhibitors have limited therapeutic efficacy [3]. In the combined glioblastoma survival network, there are 46 genes that interact with EGFR, of which ten (22%) happen to be targets of existing drugs. This observation suggests that a flanking attack strategy that strikes at both EGFR and its partner genes in the glioblastoma survival network may be an effective approach to treating these tumors.

## Conclusions

By elucidating a genetic survival network for glioblastoma, we gained insight into the mechanisms of proliferation of this cancer and opened up new avenues for therapeutic intervention.

## References

[B1] Lin A Wang RT Ahn S Park CC Smith DJ A genome-wide map of human genetic interactions inferred from radiation hybrid genotypes Genome Res 2010 20 1122 1132 10.1101/gr.104216.109 20508145 PMC2909575

[B2] The Cancer Genome Atlas Research Network Comprehensive genomic characterization defines human glioblastoma genes and core pathways Nature 2008 455 1061 1068 10.1038/nature07385 18772890 PMC2671642

[B3] Lo HW EGFR-targeted therapy in malignant glioma: novel aspects and mechanisms of drug resistance Curr Mol Pharmacol 2010 3 37 52 20030624 10.2174/1874467211003010037 PMC3606625

